# Association between vaccinations and risk of dementia: a systematic review and meta-analysis

**DOI:** 10.1093/ageing/afaf331

**Published:** 2025-11-21

**Authors:** Stefania Maggi, Tamàs Fulöp, Elda De Vita, Federica Limongi, Damiano Pizzol, Francesco Di Gennaro, Nicola Veronese

**Affiliations:** Institute of Neuroscience, National Research Council, Padova, Italy; Research Center on Aging, Université de Sherbrooke, Sherbrooke, Quebec, Canada; Clinic of Infectious Diseases, Department of Precision and Regenerative Medicine and Ionian Area (DiMePRe-J), University of Bari “Aldo Moro”, Piazza Giulio Cesare n. 11, Cap 70124 Bari, Italy; Institute of Neuroscience, National Research Council, Padova, Italy; Health Unit, ENI, San Donato Milanese, Milan, Italy; Clinic of Infectious Diseases, Department of Precision and Regenerative Medicine and Ionian Area (DiMePRe-J), University of Bari “Aldo Moro”, Piazza Giulio Cesare n. 11, Cap 70124 Bari, Italy; University of Palermo, Department of Medicine, Geriatrics Section, Palermo, Italy; Unità Locale Socio Sanitaria 3 Serenissima, Primary Care Department, Mirano-Dolo, Venice, Italy

**Keywords:** dementia, vaccination, meta-analysis, systematic review, older people

## Abstract

**Importance:**

Dementia is a highly prevalent issue in older people. Whilst the prevention of dementia is a public health priority, the role of vaccinations is still largely unexplored.

**Objective:**

The aim of this systematic review is to evaluate whether common adult vaccinations are associated with a reduced risk of dementia.

**Data sources:**

PubMed, Embase and Web of Science were searched from inception to 1 January 2025.

**Study selection:**

Observational studies comparing dementia and mild cognitive impairment incidence between vaccinated and unvaccinated adults aged ≥50 years.

**Data extraction and synthesis:**

Four reviewers independently extracted data and assessed study quality using the Newcastle–Ottawa Scale. Risk ratios (RRs) and 95% confidence intervals (CIs) were pooled using a random-effects model.

**Main outcomes and measures:**

Incidence of dementia, including its subtypes.

**Results:**

Twenty-one studies (*n* = 104 031 186 participants) were included. Vaccination against herpes zoster was associated with a reduced risk of any dementia (RR 0.76, 95% CI 0.69–0.83) and Alzheimer’s disease (RR 0.53, 95% CI 0.44–0.64). Influenza vaccination was linked to a reduction in dementia risk (RR 0.87, 95% CI 0.77–0.99), as was pneumococcal vaccination (RR 0.64, 95% CI 0.47–0.87) for Alzheimer’s disease. Tetanus, diphtheria, pertussis (Tdap) vaccination was also associated with a significant reduction for any dementia (RR 0.67, 95% CI 0.54–0.83).

**Conclusions and relevance:**

Adult vaccinations, particularly against herpes zoster, influenza, pneumococcus and Tdap, are associated with a lower risk of dementia. Vaccination strategies should be incorporated into public health initiatives for dementia prevention.

**Registration:**

https://osf.io/x3d4f/

## Key Points

- Vaccines are associated with a lower risk of dementia in adults aged 50 and older.
- Ageing immune systems benefit from routine adult vaccinations.
- Low vaccine uptake in older adults is a public health concern.
- Vaccinations support healthy ageing and dementia prevention.

## Introduction

Dementia, encompassing conditions such as Alzheimer’s disease, vascular dementia and other neurodegenerative disorders, represents one of the most pressing public health challenges globally. As the global population ages, the prevalence of dementia is projected to escalate dramatically, with an estimated 150 million people expected to be living with dementia by 2050 [1]. The growing burden of dementia is not only a personal tragedy for affected individuals and their families but also imposes substantial economic strain on healthcare systems and societies worldwide [2]. Therefore, identifying modifiable risk factors and potential preventive strategies is a public health priority.

Many studies are devoted to modulating the risk factors contributing to dementia incidence although, to date no clear evidence exists on the effect of single or multi-domain lifestyle interventions on dementia incidence [3]. Since there is large scientific support for the role of infections in the development of dementia, the possibility is raised that vaccination may be an efficient intervention to decrease the risk of dementia [4].

Recent evidence has pointed to a potentially protective role of common vaccinations in reducing the incidence of dementia and mild cognitive impairment (MCI). A range of vaccinations have been associated with a reduced risk of developing dementia. For example, our group has published a systematic review and meta-analysis on the efficacy of influenza vaccination against the onset of dementia [5]. Similarly, the vaccination against herpes zoster seems to have a similar effect on the risk of dementia [6].

Despite the emerging body of evidence supporting the neuroprotective potential of vaccines, vaccination rates amongst older adults—who are most at risk of dementia—remain suboptimal. According to the World Health Organization and national immunisation registries, influenza vaccine coverage amongst older adults rarely exceeds 60% in most high-income countries and is substantially lower in low- and middle-income settings [7]. Uptake of other vaccines such as pneumococcal or herpes zoster/shingles vaccines is even more limited, often falling below 40% [8]. In many instances, coverage for tetanus, diphtheria, pertussis (Tdap) or shingles vaccines amongst older people is less than 30%, especially when they are not included in routine adult immunisation schedules [9].

This low vaccination uptake in older populations may be driven by a combination of factors, including vaccine hesitancy, lack of awareness of adult immunisation benefits, limited access to healthcare services and absence of targeted policies promoting vaccinations beyond childhood [10]. Misconceptions about vaccine safety or efficacy, particularly in the context of ageing and polypharmacy, further exacerbate this problem [10]. Additionally, many healthcare systems fail to systematically integrate vaccination into geriatric care or dementia prevention strategies.

The low coverage is particularly concerning in light of the biological plausibility linking vaccines to reduced neuroinflammation—a key mechanism underlying neurodegeneration. Vaccinations may prime the immune system in a way that modulates long-term inflammatory responses, mitigate the effects of latent infections reactivation, reduces infection-related systemic inflammation, modulates the trained innate immunity and thus mitigates downstream effects on brain ageing and cognitive decline [11].

Given the profound impact dementia has on individuals and society, and the growing evidence of vaccination as a potential modifiable risk factor, it is crucial to promote widespread adoption of adult vaccination programmes, particularly amongst older people. Given this background, in this systematic review and meta-analysis, we have synthesised the current evidence from observational studies investigating the association between vaccination and the onset of dementia or MCI. By providing a comprehensive analysis of existing literature across multiple vaccine types, this work seeks to better understand the preventive potential of vaccines and to emphasise the urgency of improving vaccination coverage amongst older adults.

## Materials and methods

This systematic review followed a preregistered protocol (https://osf.io/x3d4f/). We followed the guidance of the Preferred Reporting Items for Overviews of Reviews statement [12].

### Data sources and searches

Four investigators (F.L., D.P., E.D.V., F.D.G.) independently conducted a literature search using Pubmed, Embase and Web of Science from database inception until 1 January 2025, including observational studies, either prospective or retrospective, investigating the effect of vaccinations on dementia and MCI onset. The search strategies included a combination of free and Medical SubHeadings (MeSH) terms following the Participants, Intervention, Comparison, Outcomes (PICO) question, i.e. vaccination, dementia and MCI, adapted for observational studies.

The full search strategies, detailed database by database, were reported in Supplementary Table S1.

### Study selection

Inclusion criteria for this meta-analysis were: (i) reporting data on any vaccination at the baseline, (ii) having a control group not taking any vaccination, (iii) reporting a validated diagnosis of the outcomes of interest (e.g. International Classification of Diseases or Diagnostic and Statistical Manual of Mental Disorders) and (iv) observational study, having a follow-up (prospective or retrospective studies). Studies were excluded if: (i) did not include humans and (ii) were cross-sectional design. The study selection for title/abstracts was performed in Rayyan [13]: all the papers were independently evaluated by two different authors (F.L., D.P., E.D.V., F.D.G.) with a senior authors resolving the conflicts (N.V.). The same process was applied at full-text level.

### Data extraction

Four independent investigators (F.L., D.P., E.D.V., F.D.G.) extracted key data from the included articles in a standardised Excel spread sheet, with a third independent investigator (N.V.) checking the data. For each article, we extracted data on authors name, year of publication, country, type of study, main condition, sample size (including information on vaccinated or not), age and percentage of females, type of vaccine, diagnostic criteria used for the definition of dementia or MCI, time of follow-up (in years) and the kind and number of confounders used in the multivariate analyses or in the propensity-score matching. The type of vaccination was then categorised in against COVID-19; herpes zoster; diphtheria, tetanus, pertussis, as single vaccinations or in combination; influenza; poliomyelitis; pneumococcus.

### Outcomes

The primary outcome was the risk of dementia, according to vaccination status, categorised as yes versus no. We considered the risk of MCI as secondary outcome. Dementia was then categorised in any dementia, Alzheimer’s disease, vascular dementia, other forms of dementia, as originally reported by the authors.

### Quality assessment

Four independent researchers (F.L., D.P., E.D.V., F.D.G.) carried out the assessment of the studies’ quality using the Newcastle–Ottawa Scale (NOS) [14]. The NOS assigns a maximum of nine points based on three quality parameters: selection, comparability and outcome. As per the NOS grading in past reviews, we graded studies as having a high (< 5 stars), moderate (5–7 stars) or low risk of bias (≥ 8 stars) [15].

### Data synthesis and analysis

The primary analysis compared the risk of dementia between participants vaccinated and those not vaccinated. We calculated the risk ratio (RR) with their 95% confidence intervals (CIs), applying a random-effects model, using the data from multivariable analyses or from propensity score analysis [16].

Heterogeneity across studies was assessed by the *I*^2^ metric and *χ*^2^ statistics. Given significant heterogeneity (*I*^2^ ≥ 50%, *P* < .05) and for outcomes having at least 10 studies, we planned to conduct a series of meta-regression analyses [17] that was possible only for the association between influenza and any kind of dementia. We used, as moderators, mean age, percentage of females and the length of the follow-up. To account for between-study effects and evaluate the likelihood of consistent results in the new studies, we calculated prediction intervals and their 95% CIs [18]. Publication bias was assessed by visually inspecting funnel plots and using the Egger bias test [19]; in case of publication bias, a fill and trim analysis was performed [20].

For all analyses, a *P*-value less than .05 was considered statistically significant. All analyses were performed using STATA version 16.0 (StataCorp).

## Results

### Literature search

**Figure 1:**
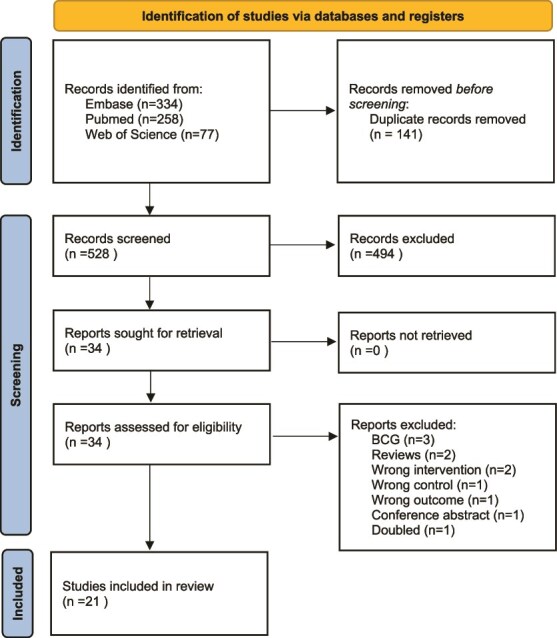
PRISMA flowchart.

Figure 1 shows the flowchart of the inclusion and exclusion of the articles of this systematic review. After removing the duplicates, 528 articles were evaluated at title/abstract level. Of them, 34 were revised at full-text level and, finally, 21 included [21–41].

### Descriptive findings

Supplementary Table S2 reports the descriptive characteristics of the 21 studies included. Overall, a total of 104 031 186 people were included: where reported, the mean age was 74 years and the majority were men (61%). Ten studies were conducted in North America, six in Europe (of them, five in the UK) and five in Asia. Amongst the studies included, only three included, at baseline, patients with particular conditions, including chronic obstructive pulmonary disease [30], periodontitis [26] and chronic kidney failure [27]. The mean follow-up period was 6.5 years, with a range between 3 months and 16 years. The details about the type of vaccinations and dementia/MCI were reported in Supplementary Table S2. Finally, the mean number of confounders included in the propensity score matching or in the multivariate analyses was 15. Although the included studies adjusted for a wide range of sociodemographic, clinical and healthcare utilisation factors—and several employed propensity score methods—the possibility of residual confounding cannot be excluded, particularly, if vaccinated individuals are generally healthier or more health-conscious. The quality of the studies, assessed with the NOS, was overall good.

### Main findings

**Figure 2:**
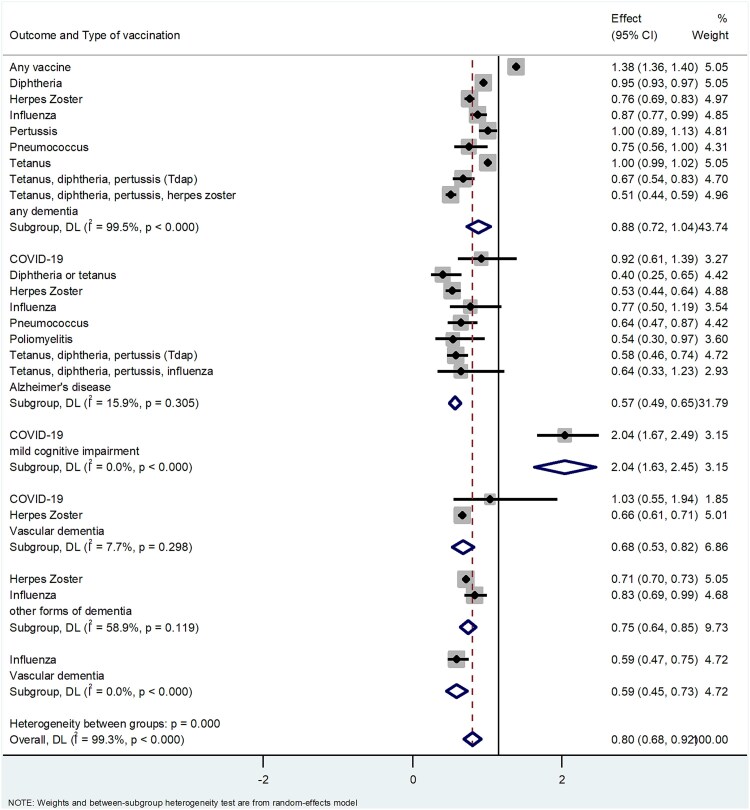
Association between vaccinations and outcomes of interest. DL, DerSimonian–Laird.

Table 1 reports the data about the potential association between the type of vaccinations and the outcomes of interest. Figure 2 shows the same association, divided by the outcomes of interest. We briefly report here the main findings of the results.

**Table 1:** Association between vaccinations and dementia and MCI.

Type of vaccination	Outcome	*N* of cohorts	*N* vaccinated	*N* controls	Type of ES	Mean ES	LL	UL	*P*	*I* ^2^	95% LLPI	95% ULPI	Publication bias
Any vaccine	Any dementia	1	652 704	194 187	RR	1.38	1.36	1.4	<.0001	NA	NA	NA	NA
COVID-19	Alzheimer’s disease	3	346 557	116 061	RR	0.92	0.61	1.39	.7	78.6	0.01	85.21	No
COVID-19	Vascular dementia	3	346 557	116 061	RR	1.03	0.55	1.94	.92	0	0.25	2.85	No
COVID-19	MCI	3	346 557	116 061	RR	2.04	1.67	2.49	<.0001	19.2	0.4	10.49	No
Herpes zoster	Any dementia	8	979 768	9 455 017	RR	0.76	0.69	0.83	<.0001	97.2	0.95	1.04	No
Herpes zoster	Alzheimer’s disease	7	1 144 479	8 728 773	RR	0.53	0.44	0.64	<.0001	99.3	0.28	1.02	No
Herpes zoster	Vascular dementia	1	155 972	180 369	RR	0.66	0.61	0.71	<.0001	NA	NA	NA	NA
Herpes zoster	Other forms of dementia	1	854 745	8 490 813	RR	0.71	0.7	0.73	<.0001	NA	NA	NA	NA
Diphtheria	Any dementia	1	58 521	788 370	RR	0.95	0.93	0.97	<.0001	NA	NA	NA	NA
Diphtheria or tetanus	Alzheimer’s disease	1	1725	2140	RR	0.4	0.25	0.65	<.0001	NA	NA	NA	NA
Tetanus	Any dementia	1	110 313	736 578	RR	1	0.99	1.02	.99	NA	NA	NA	NA
Tdap	Any dementia	4	41 170	165 109	RR	0.67	0.54	0.83	<.0001	92.7	0.25	1.84	No
Tdap	Alzheimer’s disease	3	143 880	116 669	RR	0.58	0.46	0.74	<.0001	88.7	0.03	10.56	No
Tdap, influenza	Alzheimer’s disease	2	2734	1777	RR	0.64	0.33	1.23	.18	53	NA	NA	NA
Tdap, herpes zoster	Any dementia	2	5532	164 840	RR	0.51	0.44	0.59	<.0001	0	NA	NA	NA
Influenza	Any dementia	10	2 510 058	7 547 014	RR	0.87	0.77	0.99	.03	99.5	0.55	1.38	No
Influenza	Alzheimer’s disease	4	1 717 800	8 197 135	RR	0.77	0.5	1.19	.24	99.8	0.1	6.07	No
Influenza	Other forms of dementia	3	781 913	7 261 248	RR	0.83	0.69	0.99	.049	92.5	0.08	8.53	No
Influenza	Vascular dementia	2	39 426	41 478	RR	0.59	0.47	0.75	<.0001	0	NA	NA	NA
Pertussis	Any dementia	1	1603	845 288	RR	1	0.89	1.13	.99	NA	NA	NA	NA
Pneumococcus	Alzheimer’s disease	3	276 413	402 911	RR	0.64	0.47	0.87	.004	99	0.01	33.6	No
Pneumococcus	Any dementia	5	347 657	516 049	RR	0.75	0.56	1	.05	91.5	0.27	2.09	Yes
Poliomyelitis	Alzheimer’s disease	1	1076	2789	RR	0.54	0.3	0.97	.04	NA	NA	NA	NA

ES: effect size; LL: lower limit; UL: upper limit; LLPI: lower limit prediction interval; ULPI: upper limit prediction interval

### COVID-19

About COVID-19, we found a large study made in South Korea [39] and including a total of 346 557 vaccinated versus 116 601 controls (Table 1). Overall, these authors found, after adjusting for ten potential confounders and across a follow-up period of only 3 months that the vaccination against COVID-19 was associated with a statistically significant increase in the risk of MCI of about two times, whilst no effect was observed using Alzheimer’s disease or vascular dementia as outcomes. Only the association between COVID-19 vaccination and Alzheimer’s disease suffered on a high heterogeneity (*I*^2^ = 78.6%). For these three outcomes, all the associations passed the null value for the prediction intervals, whilst none suffers on publication bias.

### Herpes zoster

The data about the vaccination against herpes zoster are reported in Table 1. In six studies [23, 28, 29, 31, 33, 36] giving data for eight cohorts and a total of 979 768 vaccinated versus 9 455 017 controls, we found that the vaccination against herpes zoster was significantly associated with a reduction in the risk of any dementia (RR = 0.76; 95% CI 0.69–0.83; *P* < .0001; *I*^2^ = 97.2%). A similar effect was found for Alzheimer’s disease [24, 28, 31, 33] for which, after including 1 144 479 vaccinated against herpes zoster and 8 728 773 controls, we observed a significant reduction of 47% in the risk of dementia (RR = 0.53; 95% CI 0.44–0.64; *P* < .0001; *I*^2^ = 99.3%). Finally, in one study, vaccination against herpes zoster reduced the risk for vascular dementia [33] and for other forms of dementia [28]. None of the outcomes reported the prediction intervals excluding the null value or publication bias.

### Tetanus, diphtheria, pertussis

The data about the vaccination against tetanus, diphtheria, pertussis were reported in Table 1 as single vaccination or as combination (Tdap) or associated with other vaccination, such as herpes zoster. When considering Tdap, we observed that in four cohorts from two large studies [24, 33] including 41 170 vaccinated versus 165 109 controls, Tdap was associated with a diminished risk of any dementia (RR = 0.67; 95% CI 0.54–0.83; *P* < .0001; *I*^2^ = 92.7%). A similar effect was observed when Tdap was associated with herpes zoster [36]. Similarly, Tdap vaccination was associated with a reduced risk of Alzheimer’s disease [32, 36] of 42% (RR = 0.58; 95% CI 0.46–0.74; *P* < .0001; *I*^2^ = 88.7%) in almost 250 000 participants. When considering as single vaccinations, the vaccination against diphtheria was significantly associated with a reduction in any dementia risk of ~5% [23] as well as the vaccination against tetanus or diphtheria with an important reduction in Alzheimer’s disease risk of 60% in one study [41].

### Influenza

The findings about influenza vaccination and dementia outcomes were reported in Table 1. In nine studies [21, 23, 25–27, 29, 30, 35, 38] with a total of 2 510 058 vaccinated against influenza and 7 547 014 controls, we found that the vaccination against influenza was associated with a decreased risk of 13% in having any dementia during the follow-up period (RR = 0.87; 95% CI 0.77–0.99; *P* = .03; *I*^2^ = 99.5%). Since this outcome was characterised by a high heterogeneity and had at least 10 studies, we did run several meta-regression analyses, but no one of these factors such as mean age (*P* = .48), percentage of females (*P* = .26) and the length of the follow-up (*P* = .21) moderated our findings. On the contrary, vaccination against influenza was not associated with a decrease in the risk of Alzheimer’s disease in four studies [22, 29, 30, 38] including 1 711 800 vaccinated and 8 197 135 controls, whilst a significant decrease in other forms of dementia was reported in three studies [28, 30, 38] (RR = 0.83; 95% CI 0.69–0.99; *P* = .049), even if marginally statistically significant, and for vascular dementia (RR = 0.59; 95% CI 0.47–0.75; *P* < .0001; *I*^2^ = 0%). All the prediction models contained null values, and none of the included outcomes suffered on publication bias.

### Pneumococcus and poliomyelitis

The vaccination against pneumococcus was also reported. In three studies [24, 34, 37] including 276 413 vaccinated against 402 911 controls, this vaccination was associated with a reduction of Alzheimer’s dementia of 36% (RR = 0.64; 95% CI 0.47–0.87; *P* = .004; *I*^2^ = 99%), whilst the risk of any dementia was marginally statistically significant (two studies [23, 25]; RR = 0.75; 95% CI 0.56–1.00; *P* = .05) (Table 1). This last outcome suffered on publication bias: however, after adjusting the estimate using the trim-and-fill analysis, the data remained unchanged.

Finally, in one study [41], the authors found that the vaccination against poliomyelitis was associated with a significant reduction in the risk of Alzheimer’s disease (RR = 0.54; 95% CI 0.30–0.97; *P* = .04).

### Global effect of vaccination on cognitive outcomes

Figure 2 shows the association between the vaccinations mentioned before and the outcomes of interest. Considering seven types of different vaccinations together, we observed that vaccination was associated with a decreased risk of Alzheimer’s disease (RR = 0.60; 95% CI 0.52–0.70; *I*^2^ = 31.1%) and vascular dementia (three types of vaccination; RR = 0.65; 95% CI 0.57–0.75; *I*^2^ = 28.1%). Similarly, nine different vaccinations were associated with a reduction in the risk of any dementia (RR = 0.86; 95% CI 0.74–1.00; *I*^2^ = 99.5%).

## Discussion

This systematic review and meta-analysis synthesised available observational data examining the association between common adult vaccinations and the risk of dementia and MCI including 21 studies and more than 100 million people. We believe that our results further strength the importance of vaccinations for healthy ageing and, in particular, for the primary prevention of dementia.

Over the past three decades, infections have increasingly been recognised as a contributor to dementia risk [42]. Pathophysiological pathways involve infections such as Herpes Simplex Virus (HSV-1), influenza, pneumonia, periodontitis, pertussis and herpes zoster inducing systemic inflammation and neuroinflammation, which, over time, accelerate neurodegenerative processes leading to dementia [43]. Chronic or severe infections, in fact, may promote glial activation, release of pro-inflammatory cytokines and blood–brain barrier dysfunction, thereby facilitating neuronal injury and cognitive decline [44].

Vaccines can mitigate this risk by preventing infections and subsequently reducing systemic and neuroinflammation [45]. Moreover, vaccines may ‘train’ the immune system, enhancing innate immune responses to clear pathogens, as well as pathological proteins such as beta-amyloid plaques, implicated in Alzheimer’s disease pathogenesis [46]. Additionally, it was reported that infections may increase the risk of vascular insults like myocardial infarction and stroke, major contributors to dementia [47]. Vaccines that reduce infection burden might therefore preserve vascular health and lower the dementia burden indirectly [48]. Finally, hospitalisations, particularly when affecting frail older adults, may accelerate cognitive decline due to immobilisation, delirium and systemic insults [49]. Since vaccination is one of the most efficient interventions to prevent severe infections and subsequent hospital admissions, they could thus contribute to better cognitive outcomes amongst older people, finally decreasing the risk of dementia.

From a quantitative point of view, our systematic review suggests that vaccination against herpes zoster, influenza, pneumococcus, diphtheria, tetanus and pertussis was associated with lower dementia risk. Notably, the vaccination against herpes zoster was able to decrease not only the risk of any dementia, but also that of Alzheimer’s disease. Our findings are in line with some recent results made in two large cohorts, unfortunately not meta-analysable, about this vaccination and the prevention of dementia. [4, 50] Similarly, we have reported that it is important to vaccinate older people for Tdap since this vaccination as well was significantly associated with an important reduction in the risk of dementia and Alzheimer’s disease. Finally, our newer results confirmed the importance of influenza vaccination to reduce the risk of overall dementia (of about 13%), as we found in another systematic review about the same topic [5].

On the contrary, the data about COVID-19 vaccinations are in contrast with those of the other vaccines mentioned before. This unexpected finding is probably due to some methodological shortcomings of the studies included. First, dementia develops over many years and vaccines for other vaccine-preventable infectious diseases have been used for decades: therefore, we have long-term data linking them to lower dementia risk, whilst COVID-19 vaccines only became widely available in 2021. Therefore, in the studies included having a follow-up of only 3 months, it is likely a reverse causation. Second, several studies show that people who’ve had COVID-19—especially severe cases—are at higher risk of cognitive decline and dementia-like symptoms, likely due to different pathophysiological mechanisms, such as neuroinflammation, vascular damage, hypoxia and autoimmune effects. Therefore, we believe that the vaccine might help protect against those effects by preventing severe COVID and long COVID-19 symptoms, but again, long-term studies are still ongoing [51, 52].

Importantly, many studies had high heterogeneity, particularly for influenza and herpes zoster vaccine analyses, and although some meta-regressions were performed, age, sex distribution or follow-up time did not significantly moderate the results.

We believe that the results of this review and meta-analysis offer several important implications, for clinicians and for public health stakeholders, also considering that dementia is still not treatable and about half of the incidence of dementia worldwide could be prevented [53]. Despite these encouraging findings, we should report several limitations. First, whilst the results suggest a potentially protective effect of several vaccines, notable methodological limitations temper these conclusions. High heterogeneity across studies was observed, driven by several factors: the diverse neuropsychological assessments used for dementia diagnosis (e.g. International Classification of Diseases (ICD codes), Diagnostic and Statistical Manual of Mental Disorders (DSM) criteria and clinical evaluations), significant variability in the adjustment for confounders (ranging from a few to dozens of variables), differences in the age at recruitment and mean age of study participants (mean age ~74 years, often with wide variability) and possible gender imbalances, with many cohorts being male-dominated. These aspects complicate the direct comparison and pooling of study results. Second, the findings were only observational and retrospective: we hope in further intervention research to consolidate our knowledge about vaccinations and cognitive aspects. Finally, some vaccinations should be repeated during the lifetime (e.g. influenza or Tdap): unfortunately, this important information was not available in the studies included. Whilst many studies adjusted for sociodemographic, comorbidities and healthcare utilisation—and some used propensity score matching—it remains possible that vaccination status reflects broader differences in health behaviours, access to care or cognitive reserve that were not fully captured. Thus, healthy vaccine bias cannot be entirely excluded and should be considered when interpreting the observed associations.

In conclusion, adult vaccinations, particularly against herpes zoster, influenza, pneumococcus and Tdap, are associated with a lower risk of dementia. Therefore, our systematic review indicates that vaccination strategies should be incorporated into public health initiatives for dementia prevention. However, high heterogeneity and observational designs underscore the need for prospective trials.

## Supplementary Material

Supplementary_materials_afaf331
