# Supplementary material for: Association between vaccinations and risk of dementia: a systematic review and meta-analysis
Source: Age Ageing. 2025 Nov 21;54(11):afaf331. doi: 10.1093/ageing/afaf331 (PMC12636520; doi:10.1093/ageing/afaf331)
Supplement: Supplementary_materials_afaf331 [file supplementary_materials_afaf331.zip › Supplementary_materials_afaf331.docx]

**Supplementary Table 1. Search strategy**

| **Database** | **Search strategy** | **#** |
| --- | --- | --- |
| **Pubmed** | ("Vaccines"[Mesh] OR vaccin* OR vaccine* OR "Vaccination") AND ("Dementia"[Mesh] OR "Amentia" OR dementia OR demention OR "Alzheimer Disease"[Mesh] OR "Alzheimer Dementia" OR "Alzheimers Disease" OR "Alzheimer's Disease" OR Multi-Infarct Dementia OR Multiinfarct Dementia OR "Vascular Dementia" OR "Lewy Body Disease"[Mesh] OR "Diffuse Lewy Body Disease" OR "Lewy Body Dementia" OR "Dementia with Lewy Bodies" OR "Frontotemporal Dementia"[Mesh] OR "Frontal Lobe Dementia" OR "Frontotemporal Dementias" OR "Pick's Complex") AND ("Cohort Studies"[Mesh] OR "Longitudinal Studies"[Mesh] OR "Cohort Study" OR "Cohort Analysis" OR "Longitudinal Study" OR "Longitudinal Evaluation" OR longitudinal OR cohort OR retrospective) | 258 |
| **WoS** | TS=("vaccine*" OR "combined vaccine*" OR vaccin* OR "vaccine control" OR "vaccine efficacy" OR "vaccine potency" OR "vaccine safety" OR "vaccination" OR "vaccination policy" OR "vaccination program*" OR vaccinotherapy OR "virus vaccination") AND TS=("dementia" OR "amentia" OR "demention" OR "alzheimer disease" OR "alzheimer's disease" OR "alzheimer dementia" OR "alzheimer fibrillary change" OR "alzheimer neurofibrillary degeneration" OR "alzheimer syndrome" OR "cortical sclerosis, diffuse" OR "multi-infarct dementia" OR "vascular dementia" OR "lewy body dementia" OR "dementia with lewy bodies" OR "frontotemporal dementia" OR "frontal lobe dementia" OR "pick's complex") AND TS=("cohort analysis" OR "cohort study" OR "cohort studies" OR "longitudinal study" OR "longitudinal studies" OR “retrospective”) | 77 |
| **Embase** | ('vaccine'/exp OR 'combined vaccine' OR 'vaccin' OR 'vaccine' OR 'vaccine control' OR 'vaccine efficacy' OR 'vaccine potency' OR 'vaccine safety' OR 'vaccines' OR 'vaccines, combined' OR 'vaccination'/exp OR 'vaccination' OR 'vaccination policy' OR 'vaccination program' OR 'vaccination programme' OR 'vaccinotherapy' OR 'virus vaccination') AND ('dementia'/exp OR 'amentia' OR 'dementia' OR 'demention' OR 'alzheimer disease'/exp OR 'alzeimer disease' OR 'alzeimer`s disease' OR 'alzeimers disease' OR 'alzheimer dementia' OR 'alzheimer disease' OR 'alzheimers disease' OR 'alzheimer fibrillary change' OR 'alzheimer fibrillary lesion' OR 'alzheimer neurofibrillary change' OR 'alzheimer neurofibrillary degeneration' OR 'alzheimer neuron degeneration' OR 'alzheimer perusini disease' OR 'alzheimer sclerosis' OR 'alzheimer syndrome' OR 'alzheimer`s disease' OR 'cortical sclerosis, diffuse' OR 'dementia, alzheimer' OR 'diffuse cortical sclerosis' OR 'late onset alzheimer disease' OR 'multiinfarct dementia'/exp OR 'dementia, multi-infarct' OR 'dementia, multiinfarct' OR 'dementia, vascular' OR 'lacunar dementia' OR 'multi-infarct dementia' OR 'multi-infarction dementia' OR 'multiinfarct dementia' OR 'multiinfarction dementia' OR 'vascular dementia' OR 'diffuse lewy body disease'/exp OR 'dlb (dementia with lewy bodies)' OR 'dlbd' OR 'lbd (lewy body disease)' OR 'lewy body dementia' OR 'lewy body dementias' OR 'lewy body disease' OR 'lewy body diseases' OR 'dementia with lewy bodies' OR 'dementia with lewy body' OR 'diffuse lewy body disease' OR 'frontotemporal dementia'/exp OR 'ftd (frontotemporal dementia)' OR 'ftld' OR 'pick complex' OR 'pick`s complex' OR 'dementia, frontotemporal' OR 'frontal dementia' OR 'frontal lobe dementia' OR 'frontotemporal dementia' OR 'frontotemporal dementias' OR 'frontotemporal lobar degeneration') AND ('cohort analysis'/exp OR 'analysis, cohort' OR 'cohort analysis' OR 'cohort fertility' OR 'cohort life cycle' OR 'cohort studies' OR 'cohort study' OR 'fertility, cohort' OR 'longitudinal study'/exp OR 'longitudinal evaluation' OR 'longitudinal studies' OR 'longitudinal study' OR 'retrospective ' ) | 334 |

**Supplementary Table 2. Descriptive characteristics of the studies included**

| **Author, year** | **Country** | **Continent** | **Type of study** | **Condition** | **Sample size** | **Follow-up length (years)** | **Mean age** | **SD age** | **% of females** | **Type of vaccine** | **Definition of dementia-outcome** | **Confounders** | **N of confounders** | **NOS** |
| --- | --- | --- | --- | --- | --- | --- | --- | --- | --- | --- | --- | --- | --- | --- |
| Appel, 2024 | Denmark | Europe | Retrospective | Older people | 1673421 | 16 | 82.9 vaccinated; 81.6 non vaccinated | (78.1. 87.4) vaccinated; 75.7. 86.6 non vaccinated | 53.6 vaccinated; 53.8 non vaccinated | Influenza | ICD-8/ICD-10- AD, vascular dementia, frontotemporal dementia, dementia without specification, other dementias | age, sex, calendar year, marital status, educational attainment,hypertension, hyperlipidaemia, Peripheral vascular disease, stroke, cardiac arrhythmia,diabetes,parkinson, depresson, alcohol abuse | 14 | 9 |
| Bukhbinder, 2022 | USA | north america | Retrospective | general Population | 2356479 | 4 | 73 | 6 | 57% vaccinated 56.8 %non vaccinated | Influenza | ICD 9- AD | age, sex, comorbidities, medicaments, geographic region, No. of healthcare visits during look-back,No. of routine “well visit” examinations | 8 | 6 |
| Douros, 2022 | UK | europe | Retrospective | General Population > 50 y | 13383431 | 10.3 | 70 | 10 | 62% | any vaccine, diphteria, Herpes Zoster, Influenza, pertussis, pneumococcus, tetanus | Clinical Practice Research Datalink - Any dementia | body mass index, smoking, socioeconomic status, ethnicity, alcohol-related disorders, arterial hypertension, atrial fibrillation, congestive heart failure, coronary artery disease, stroke or transient ischemic attack, peripheral vascular disease, dyslipidemia, diabetes mellitus, chronic kidney disease, liver disease, depression, epilepsy, Parkinson disease, traumatic brain injury, osteoporosis, hypothyroidism, cancer, treatment for varicella zoster virus infection, antibiotics, oral anticoagulants, antiplatelet agents, lipid-lowering drugs, β-blockers, thiazides, angiotensin-converting enzyme inhibitors, angiotensin II receptor blockers, calcium channel blockers, proton pump inhibitors, nonsteroidal anti-inflammatory drugs, opioids, immunosuppressants and biologics, antipsychotics, and antidepressants. | 38 | 8 |
| Harris,2023 | USA | America | Retrospective | general population | 1651991 | 8 |  |  |  | Herpes Zoster, pneumococcus, tetanus, diphtheria, pertussis (Tdap) | ICD9 - AD | age, sex, race, geographical region, Number of healthcare encounters, Number of routine “well visit” examinations, comorbidities, medications | 24 | 8 |
| Xingyue HUO, 2024 | USA | north america | Retrospective | general population | 157269 | 3 | 74.7 | 5.67 | 55 | pneumococcus | ICD9 - AD | sex,age, comorbidities | 3 | 8 |
| Kousuke Iwai-Saito,2023 | Japan | Asia | prospective | older people | 9865 | 3.5 | 74 | 6.26 | 53.9 | Influenza, pneumococcus | Ministry of Health, Labour and Welfare criteria - Any dementia | age, sex,socioeconomic status (SES), health behaviors, BMI, chronic diseases,geriatric factors, history of vaccination and infection, social capital | 10 | 7 |
| Lee CY, 2020 | Taiwan | Asia | case-control | patients with periodontitis | 112036 | 7.5 | ≥50 years | not reported | 51.6 | Influenza | ICD-9 - any dementia | age, sex, low income, hypertension, mental disorders, diabetes, ischemic Ischaemic heart disease, Stroke, Hyperlipidaemia, COPD, heart failure, Liver cirrhosis, Traumatic brain, Renal dialysis, influenza vaccination, use of statins, use of metformin, Time period of follow-up, | 18 | 9 |
| Liu JC, 2016 | Taiwan | Asia | prospective | patients with chronic kidney disease | 11943 | 7 | 72.95 | 7.98 | 43.22 | Influenza | ICD-9 - any dementia | age, sex, diabetes, hypertension, dyslipidemia, cerebrovascular diseases, parkinsonism, epilepsy, substance- and alcohol-use disorders, mood disorder, anxiety disorder, psychotic disorder, sleep disorder, level of urbanization, monthly income in propensity score. | 16 | 9 |
| Lophatananon A, 2023 | UK | Europe | Retrospective | older people | 83235813 | 3.1 | ≥ 70 | not reported | 52.3 | Herpes Zoster, influenza | ICD-10 - Any dementia, AD, other forms | gender, age, practice-level factors, IMD, ethnicity, time-dependent indicators for the conditions in the Charlson Comorbidity Index, time-dependent antiviral status | 7 | 7 |
| Lophatananon A, 2021 | UK | Europe | nested case–control | General population | 228223 | 3 | cases= 68.91; controls=65.35 | cases=6.51; controls=8.07 | 54.41 | Herpes Zoster | ICD-10, ICD-9 - any dementia | age, age at vaccination, sex, prior shingles diagnosis, Charlson Comorbidity Index | 5 | 7 |
| Luo CS, 2020 | Taiwan | Asia | Retrospective | patients with COPD | 19848 | 7.4 | 71.9 | 7.94 | 42.16 | Influenza | ICD-9-CM - Any dementia, vascular dementia, AD | COPD-related hospitalization, age, sex, diabetes, hypertension, dyslipidemia, cerebrovascular diseases, parkinsonism, epilepsy, substance use and alcohol disorder, mood disorder, anxiety disorder, psychotic disorder, sleep disorder, level of urbanization, monthly income | 17 | 9 |
| Roh JH, 2024 | Seoul, South Korea | Asia | Retrospective | city residents | 558017 | 0.25 | 72.90 | 6.57 | 52.38 | COVID-19 | ICD-10 - AD, vascular dementia, MCI | gender, age, insurance level, Charlson comorbidity index, diabetes mellitus, hypertension, hyperlipidemia, chronic obstructive, pulmonary disease, history of previous COVID-19 infection | 10 | 8 |
| Scherrer JF, 2021 | USA | North America | Retrospective | MarketScan patients | 17279 | 3.1 | 69.9 | 5.7 | 65 | Herpes Zoster | ICD-9, ICD-10 - any dementia, AD | age, gender, region, health services utilization, number of well visits, type 2 diabetes, obesity, hypertension, Hyperlipidemia, stroke, ischemic heart disease, congestive heart failure, atrial fibrillation, asthma, COPD, traumatic brain injury, Vitamin B12 deficiency, depression, anxiety disorder, nicotine dependence, alcohol and drug abuse/dependence, Anticholinergics, NSAIDs, antihypertensives, Statins, glucocorticoids, antivirals, metformin, sulfonylurea, Year of cohort entry, HZ infection, HZ antiviral | 32 | 9 |
| Scherrer JF, 2021 | USA | North America | Retrospective | Veterans Health Administration (VHA) patients | 122946 | 7.92 | 75.6 | 7.5 | 4 | tetanus, diphtheria, pertussis (Tdap) | ICD-9/ICD-10 - any dementia, AD | age, race, gender, marital status, VA only health insurance vs. VA+private/Other, Region, High health services utilization, Number of well visits, type 2 diabetes, obesity, hypertension, stroke, ischemic heart disease, congestive heart failure, atrial fibrillation, asthma, COPD, traumatic brain injury, Vitamin B12 deficiency, depression, anxiety disorder, nicotine dependence, alcohol and drug abuse/dependence, anticholinergics, NSAIDs, statins, steroids, antivirals, metformin, sulfonylurea, Year of cohort entry | 31 | 9 |
| Schnier, 2022 | UK | Europe | Retrospective | General Population | 336341 | 6 |  |  | 51.8 | Herpes Zoster | British RCHD - any dementia, AD, vascular | sex, birth year, care home, prior vaccination, diabetes, cancer, CVD, COPD, chronic heart disease, chronic liver disease, myocardial infraction, peptic ulcer, perivascular disease, renal disease, rheumatic disease | 15 | 8 |
| Tyas, 2001 | Canada | America | Retrospective | General Population | 626 | 5 | 74 | 5.8 | 62.4 | Influenza, tetanus, polio, diphteria | NINCDS-ADRDA - AD | age, sex, education | 3 | 7 |
| Ukraintseva, 2023 | USA | America | Retrospective | General Population | 5599 | 10 |  |  | 56.3 | pneumococcus | ICD-9-CM - AD | sex, race, birth cohort, education, and smoking | 5 | 8 |
| Verrault, 2001 | Canada | America | Retrospective | General Population | 3865 | 5 | NA | NA | NA | Diphteria or tetanus, tetanus, diphtheria, pertussis (Tdap), Poliomelytis | DSM-III-R - AD | age, sex, education, current smoking, regular alcohol consumption, family history of dementia, activities of daily living and instrumental activities of daily living, antecedents of chronic diseases and perceived health status | 10 | 9 |
| Wiemken, 2021 | USA | America | Retrospective | Veterans | 123747 | 7,5 | 75.5 | 7.3 | 3.8 | Influenza | ICD-9/ICD-10 - any dementia | Not specified | 0 | 7 |
| Wiemken, 2021 | USA | America | Retrospective | Veterans | 74455 | 7 | 76.8 | 7.6 |  | Herpes Zoster, tetanus, diphtheria, pertussis (Tdap), tetanus, diphtheria, pertussis (Tdap) and herpes zoster | ICD-9/ICD-10 - any dementia | anti-hypertensive medication adherence, defined as ≥80% of proportion of days covered, neighborhood socioeconomic inde | 2 | 7 |
| Zhao H, 2024 | UK | Europe | Retrospective | community-dwelling adults | 70938 | 12.2 | 64.4 | 3.1 | 53.6 | Influenza | ICD-9/ICD-10 - any dementia, AD, vascular dementia , other forms | sex, age, education, Townsend deprivation index (TDI), average household income, region of assessment center, self-reported health rating, family history of dementia/PD, BMI, mental health score, smoking status, drinking status, diet, tea and coffee intake, physical activity, social isolation, ApoE genotype, FluVac invitation, comorbidities, aspirin, glucose-lowering agents, statins, CCB, ACEI, ARB, PPI, BBA, diuretics | 29 | 8 |

Abbreviations
